# Growth Inhibition and Induction of Apoptosis in SHG-44 Glioma Cells by Chinese Medicine Formula “Pingliu Keli”

**DOI:** 10.1155/2011/958243

**Published:** 2010-09-08

**Authors:** Peng Cao, Xueting Cai, Wuguang Lu, Fei Zhou, Jiege Huo

**Affiliations:** Laboratory of Cellular and Molecular Biology, Jiangsu Province Institute of Traditional Chinese Medicine, no. 100, Shizi Street, Hongshang Road, Nanjing, Jiangsu 210028, China

## Abstract

The present study was carried out to evaluate the effects of the water extract of Chinese medicine “Pingliu Keli” (PK) on human glioma cell viability and apoptosis and to investigate its mechanisms of action in SHG-44 cells. MTT assay showed that PK had a strong cytotoxic effect on SHG-44 cells. The number of live cells was less than 20% after exposure to 90 *μ*g/mL PK for 24 h. PK increased cytotoxicity of SHG-44 cells in a dose-dependent manner. PK caused arrest of SHG-44 cells in G1 phase at low concentration and in G2 phase at high concentration. The percentage of apoptotic cells by flow cytometric analysis of the DNA-stained cells increased to 38% and 52% after treatment with 72 and 108 *μ*g/mL PK, respectively. In addition, PK increased the expression of proapoptotic protein (Bax) and decreased antiapoptotic protein (Bcl-2), with a concomitant increase in the levels of cleaved caspase-3, cleaved caspase-9 and cleaved poly-ADP-ribose polymerase (PARP). These results suggest that PK has a significant apoptosis inducing effect on SHG-44 glioma cells *in vitro* and caspase-3 may act as a potential mediator in the process.

## 1. Introduction

Up to 65% of primary brain tumors are of glial origin and these glial-derived tumors are collectively called gliomas. Glioma is one of the most malignant human tumors [1] and, despite aggressive surgical resection and radiotherapy, the median survival in these patients does not normally exceed one year [1, 2]. The use of systemic chemotherapy may improve the efficacy of treatment, but its use is associated with significant toxicity and the long-term prognosis remains poor [3]. It is now being increasingly recognized that intervening critical processes of cancer growth and development with naturally occurring herbal and phytochemical agents to achieve chemoprevention is crucial to decreasing the morbidity and mortality of these and other cancers. Chinese herbal medicine has long been used for treating malignancies [4, 5]. Whereas single herbs are seldom used alone, herbal cocktails take advantage of synergy and interactions among a myriad of phytochemicals present in the different herbs to achieve therapeutic efficacy targeting multiple biological and pathological processes while minimizing side effects [6, 7]. However, herbal remedies are yet to be integrated into main stream medicine due to a number of challenges, including herbal standardization and quality control issues, safety and toxicity concerns, interactions with existing therapeutic modalities, a lack of proven efficacy by standard clinical trials and a lack of mechanistic details, to name a few [8, 9]. Rigorous *in vitro* and preclinical animal studies will be essential and necessary to evaluate their efficacy and safety before clinical trials can be contemplated for the chemoprevention and treatment of these major cancers in humans and to transform traditional herbal practices into “evidence-based medicine”.

In China, the water decoction of “Pingliu Keli” (PK) is employed as a folk remedy for the treatment of glioma [10]. The present study examined the antiproliferative activity of a water extract of PK and its effect on the cell cycle and apoptosis of SHG-44 glioma cells. Furthermore, the levels of several important genes that are strongly associated with the signal transduction pathway of apoptosis were measured to establish the anticancer mechanism of PK.

## 2. Methods

### 2.1. Materials

DMEM medium, heat-inactivated fetal bovine serum (FBS), penicillin, and streptomycin were purchased from Gibco, USA. MTT [3-(4,5-dimethylthiazol-2-yl)-2,5-diphenyltetrazolium bromide], DMSO (dimethyl sulfoxide) were obtained from Sigma, USA. Lysis buffer was purchased from Beyotime, China. Hoechst 33258 was purchased from KeyGEN, China. FITC Annexin V Apoptosis Detection Kit was purchased from BD Biosciences, USA. Antibodies (caspase-3, caspase-9, goat antimouse IgG-HRP, and goat antirabbit IgG-HRP) were obtained from Santa Cruz, USA. Bcl-2, Bcl-X_L_, Bax, PARP antibodies was purchased from Cell Signaling Technology, USA. monoclonal mouse anti-glyceraldehyde-3-phosphate dehydrogease (GAPDH) was obtained from KangChen, China.

### 2.2. Formula Preparation

PK is composed of *Lycium chinense* (40 g), *Dendrobium officinale* (40 g), *Arisaema heterophyllum* (40 g), *Rhizoma typhonii* (40 g), *Curcuma zedoaria * (40 g), *Ligusticum chuanxiong* (40 g), *Buthus martensii Karsch *(40 g), *Bombyx mori L* (40 g), and *Herba hedyotis diffusae* (40 g). All medicinal plants used to prepare formulae were provided by Jiangsu Province Integrated Chinese and Western Medicine Hospital (Nanjing, China), plant parts, and origin used in the formula as Table 1. The plant were homogenized with a waring blender, then soaked in 10 Liter double distilled water (DDW) for 24 h. The mixture was heated to 100°C for 2 h, and the decoction was filtrated. The filtrates obtained from 3 cycles of the procedures were mixed, concentrated by heating and granulated by lyophilization. Total yield of the PK extract is 95 g lyophilized powder from water extract of 1 kg raw mixed herb. PK and its preparations were standardized, regulated, and quality-controlled according to the guidelines defined by Chinese State Food and Drug Administration (SFDA).

### 2.3. Cell Culture and Morphological Assessment

The SHG-44 human glioma cell line was obtained from Cell Bank of Shanghai Institute of Biochemistry and Cell Biology (Shanghai, China) and cultured in DMEM medium supplemented with 10% heat-inactivated fetal bovine serum (FBS), 100 U/mL penicillin and 100 *μ*g/mL streptomycin at 37°C and 5% CO_2_. SHG-44 cells were cultured in DMEM medium till mid-log phase. Distilled water (control), 36, 54, 7290 or 108 *μ*g/mL PK was then added to the culture medium. The morphology of cells was monitored under an inverted microscope (Zeiss Axio Observer A1) at 6, 12, 18, 24, and 48 h.

### 2.4. Hoechst 33258 Staining

Hoechst 33258 staining was used to visualize nuclear changes and apoptotic body formation. At the end of PK (36, 54, 72, 90, or 108 *μ*g/mL) treatment, attached cells were washed twice with PBS and fixed with 4% methanal at 4°C for 30 min. The Fixing solution was removed and cells were washed twice with PBS before staining with Hoechst 33258. After staining for 10 min, cells were washed again and observed under a fluorescence microscope (Zeiss Axio Observer A1) at 340 nm.

### 2.5. Cell Viability Assay

MTT was used as an indicator of cell viability as determined by its mitochondrial-dependent reduction to formazone. SHG-44 cells were cultured in DMEM medium till mid-log phase, then seeded in 96-well plate at a density of 1 × 10^4^ cells per well in 100 *μ*L medium. After 24 h of incubation, cells were exposed to distilled water (control), 18, 36, 54, 72, 90, or 108 *μ*g/mL PK for 24 h. After treatment, 10 *μ*L of 5 mg/mL MTT was added and the cells were incubated further for 4 h at 37°C. The supernatant was discarded and 100 *μ*L of DMSO was added to each well. The mixture was shaken on a minishaker at room temperature for 10 min and the spectrophotometric absorbance was measured by Multiskan Spectrum Microplate Reader (Thermo) at 570 nm and 630 nm (absorbance 570 nm, reference 630 nm). Triplicate experiments were performed in a parallel manner for each concentration point and the results were presented as mean ± SD. The net *A*_570 nm_ − *A*_630 nm_ was taken as the index of cell viability. The net absorbance from the wells of cells cultured with 0.1% DMSO was taken as the 100% viability value. The percent inhibition of the treated cells was calculated by the following formula:

(1) $$\begin{array}{cc} & \text{\%Inhibition}\\ & \;\;\;\;\;=\frac{\left[{\left(A_{570\;\text{nm}}-A_{630\;\text{nm}}\right)}_{\text{control}}-{\left(A_{570\;\text{nm}}-A_{630\;\text{nm}}\right)}_{\text{treated}}\right]}{{\left(A_{570\;\text{nm}}-A_{630\;\text{nm}}\right)}_{\text{control}}}\\ & \;\;\;\;\;\;\times 100\text{\%}. \end{array}$$

### 2.6. Cell-Cycle Analysis

SHG-44 Cells were plated in six-well plates at a density of 300,000 per well and allowed to adhere overnight. Cells were treated with distilled water (control), 36, 72 or 108 *μ*g/mL PK in serum-free medium for 24 h or 36 h. Cells were then trypsinized and fixed in ice cold 70% ethanol and stained with propidium iodide in PBS. Flowcytometric data were acquired using a FACSCan analysis system equipped with a FACStation, MAC PowerPC computer and CellQuest Acquisition software from Becton Dickinson (FACSCalibur, Becton Dickinson, USA).

### 2.7. Annexin-V/PI Double-Staining Assay

SHG-44 cells were treated with distilled water (control), 72 or 108 *μ*g/mL PK for 24 h. Then they were harvested, washed, and resuspended with PBS. Apoptotic cells were determined with an FITC Annexin V Apoptosis Detection Kit (BD Biosciences, USA) according to the manufacturer's protocol. Briefly, the cells were washed and subsequently incubated for 15 min at room temperature in the dark in 100 *μ*L of 1x binding buffer containing 5 *μ*L of Annexin V-FITC and 5 *μ*L of propidium iodide (PI). Afterward, apoptosis was analyzed by fluorescence microscope (Zeiss Axio Observer A1) and FACScan laser flow cytometer (FACSCalibur, Becton Dickinson, USA). 

### 2.8. Western Blotting Analysis

SHG-44 cells were cultured in DMEM till mid-log phase and then incubated with distilled water (control), 36, 72, or 108 *μ*g/mL PK for 24 h. Proteins were isolated by lysis buffer (Beyotime, China) and measured using the Nanodrop 1000 Spectrophotometer (Thermo, USA). Protein samples were separated on 13% SDS-polyacrylamide gel (SDS-PAGE) and transferred onto the PVDF membranes (Millipore). Immune complexes were formed by incubation of the proteins with primary antibodies, Bcl-2, Bcl-X_L_, Bax, PARP antibodies (Cell Signaling Technology, USA), caspase-3, caspase-9 antibodies (Santa Cruz, USA), and monoclonal mouse anti-glyceraldehyde-3-phosphate dehydrogease(GAPDH) (KangChen, China) overnight at 4°C. Blots were washed and incubated for 1 h with goat antimouse IgG-HRP or goat antirabbit IgG-HRP second antibodies (Santa Cruz, USA). Immunoreactive protein bands were detected with Gel/Chemi Doc System (Bio-rad, USA).

### 2.9. Statistical Analysis

Values were expressed as means ± standard  deviations. Value of <0.05 was considered statistically significant.

## 3. Results

### 3.1. Cell Morphological Assessment Effect of PK on Cell Morphology

**Figure 1:**
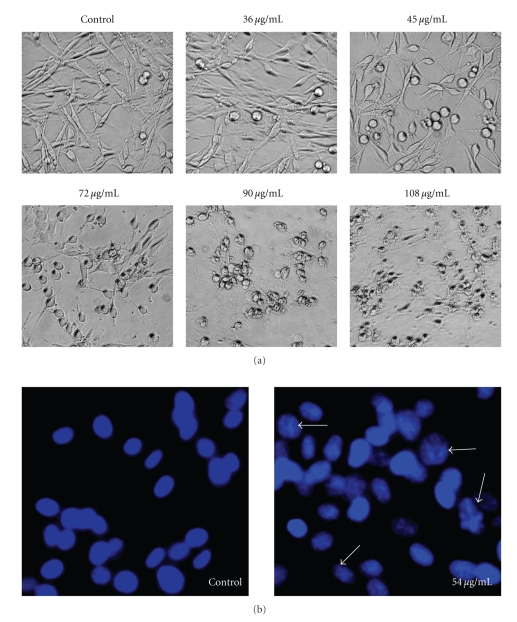
Inhibition of the cell growth and induction of apoptosis by PK in human malignant glioma cell SHG-44. After treating with different concentration of PK for 48 h, the cells were observed using an inverted microscope (Zeiss Axio Observer A1) or stained with Hoechst 33258, and then photographed with a fluorescence microscope. Compared to the control cells, cells exposed to PK presented typical apoptotic morphology with cell shrinkage, nuclear condensation, and formation of apoptotic bodies, with rupture of cells. The Hoechst 33258 dye stained morphologically normal nuclei dimly blue, whereas PK-treated cells demonstrated smaller nuclei with brilliant blue staining (arrows indicate condensed nuclei).

Differences in cell morphology were observed between PK-treated and control cells by light microscopy. The most conspicuous changes observed in PK-treated cells included cell shrinkage and extensive detachment of the cells from the cell culture substratum. These changes, which were characteristic of cell apoptotic death, became visible after 12 h of PK treatment, but were absent in control cells. The morphological changes became more remarkable with increased time of drug treatment. These observations suggested that cells treated with PK detached from the substratum and died by apoptosis (Figure 1(a)).

The occurrence of apoptosis was further verified by Hoechst staining, which detects chromatin condensation, one of the hallmarks of apoptotic cell death. Some differences were observed in the nuclei of PK-treated and -untreated SHG-44 cells after staining with Hoechst 33258 (Figure 1(b)). The Hoechst 33258 dye stained morphologically normal nuclei dimly blue whereas PK-treated cells demonstrated smaller nuclei with brilliant blue staining. The changes in nuclear morphology were initially observed after 24 h of PK treatment and increased thereafter. These results demonstrate that PK induces morphological changes characteristic of apoptotic cell death.

### 3.2. Effects of PK on the Cell Proliferation of SHG-44

**Figure 2:**
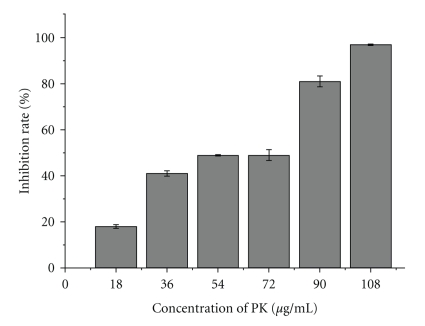
Inhibitory effect of PK on the cell proliferation of SHG-44 cells. The results shown were the mean of three parallel experiments (triplicate wells) for each concentration point (18, 36, 54, 72, 90, or 108 *μ*g/mL) (*P* < .05).

To test the effect of PK on the proliferation of SHG-44 cells, the cells were treated with different concentrations of PK. After 48 h incubation, the cell viability was measured by MTT assay. PK treatment significantly inhibited the growth of SHG-44 cells, the number of viable cells decreases as the concentration of PK increases (Figure 2). The effect of PK treatment is statistically significant when compared with the control group (*P* < .05).

### 3.3. Different Effects of Low and High Concentration of PK on SHG-44 Cell Cycle

**Figure 3:**
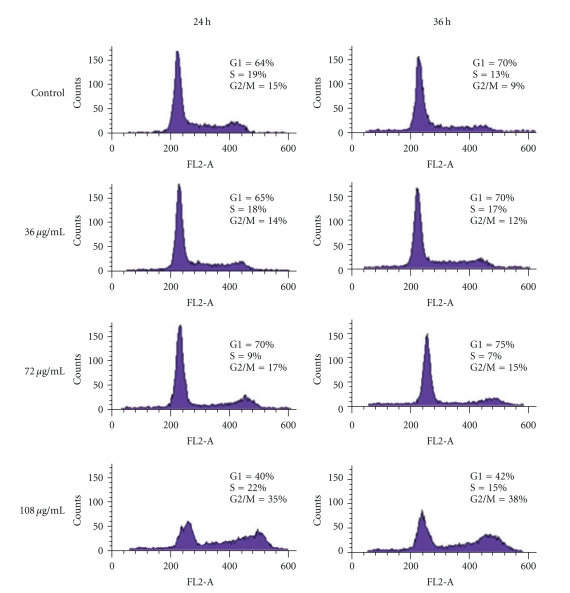
Representative histograms depicting cell-cycle distribution in SHG-44 cell cultures treated with distilled water (control) or various concentrations of PK for different period of time. 72 *μ*g/mL PK induced G1 phase arrest and reduced S phase whereas most cells were arrested at G2/M phase at 108 *μ*g/mL. All data presented were representatives of at least three independent experiments, *P* < .05.

Analysis of cell-cycle phase distribution was carried out to study the antiproliferative mechanism of PK. Different concentration of PK has different effects on SHG-44 cell-cycle. At lower doses of PK (72 *μ*g/mL), the number of SHG-44 cells during G0/G1 was increased, and the S phase was reduced dramatically. However, when the concentration was up to108 *μ*g/mL, most cells were arrested at G2/M phase. The cell-cycle arrest was evident at 24 h treatment, and many cells died after 36 h treatment of high concentration of PK. These results indicated that PK could inhibit SHG-44 cells synthesizing DNA at low concentration, whereas high concentration of PK induced G2 arrest by inhibiting DNA synthesis and cell division (Figure 3).

### 3.4. Quantification of Apoptosis by Flow Cytometry with Annexin V/PI Staining

**Figure 4:**
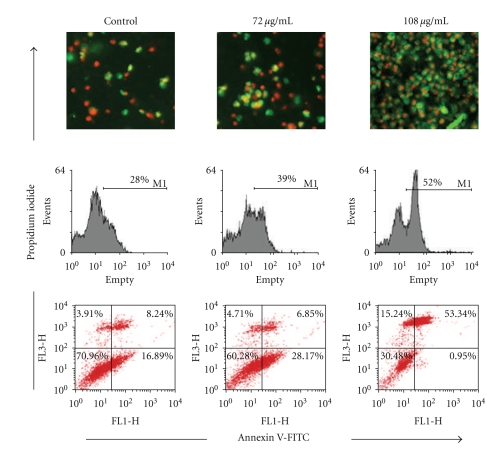
Annexin-V/PI double-staining assay. After treating with PK, cells were stained with annexin V-FITC and propidium iodide and analyzed by fluorescence microscopy. Flow cytometric analysis of annexin V-FITC/PI double-staining: SHG-44 cells were left untreated or treated for 24 h with 72 or 108 *μ*g/mL PK. Cells were incubated with Annexin V-FITC in a buffer containing propidium iodide (PI) and analyzed by flow cytometry. Untreated cells were primarily Annexin V-FITC and PI negative, indicating that they were viable and not undergoing apoptosis. After treatment, there were primarily three populations of cells: cells that were viable and not undergoing apoptosis (Annexin V-FITC and PI negative), cells undergoing apoptosis (Annexin V-FITC positive and PI negative) and population of cells were observed to be Annexin V-FITC and PI positive, indicating that they were in end stage apoptosis or already dead. (red: stained with Annexin V-FITC, green: stained with PI, mixture: stained with Annexin V-FITC and PI both).

Additional evidence for the occurrence of apoptosis was obtained by double staining of the cultures with propidium iodide (which stains the nuclei of dead cells) and annexin V-FITC, a protein that binds with high affinity to phosphatidylserine, which is translocated from the inner to the outer membrane leaflet early in the apoptotic process. Control cells stained negative for both propidium iodide and annexin V-FITC. PK-induced cells, on the other hand, showed many annexin V-positive cells. The majority of the cells were negative for propidium iodide, indicating that they were at an early stage of apoptosis. The double positive staining of particular cells revealed that these cells were at a late apoptotic (or necrotic) stage. Take together, these findings provide strong evidence that PK induced cell death through apoptosis (Figure 4).

### 3.5. Western Blot Analysis of PARP, Caspase-3, Caspase-9, Bax, and Bcl-2/*X*_*L*_ Protein

**Figure 5:**
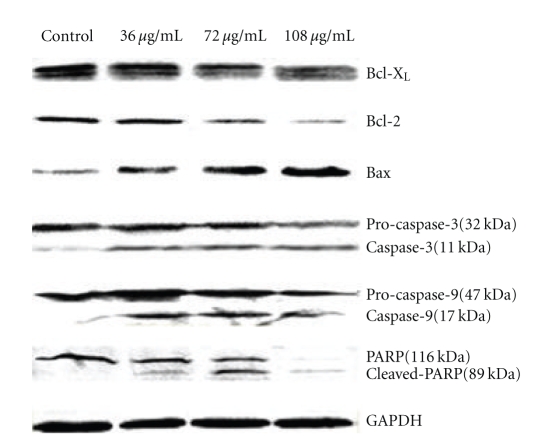
Expression of apoptosis-related proteins in PK-treated cells. The left panel denotes the effect of distilled water (control) and the right panel represents the results of 36 *μ*g/mL, 72 *μ*g/mL and 108 *μ*g/mL PK after exposure for 48 h. Data shown were representatives of at least three independent experiments.

**Figure 6:**
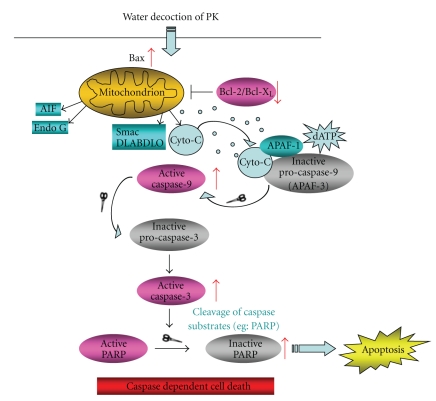
Regulation of the intrinsic apoptotic pathway by PK. The active compounds of PK reduced their effectiveness after digesting and absorbing by patients. PK increased the expression of proapoptotic protein (Bax) and decreased antiapoptotic protein (Bcl-2, Bcl-X_L_), with a concomitant increase in the levels of caspase-3, caspase-9 and cleaved poly-ADP-ribose polymerase (PARP) in SHG-44 cells. Caspase dependent cell death was the possible mechanism of PK's antitumor activity.

After exposure with PK at different concentration for 48 h, total cell lysate was prepared and an equal amount of protein was subjected to SDS-PAGE. Western blot analyses were done with anti-PARP, -caspase-3, -caspase-9, Bcl-2, Bcl-X_L_, Bax, and GAPDH primary antibodies as described in materials and methods. PK increased the expression of proapoptotic protein (Bax) and decreased antiapoptotic protein (Bcl-2, Bcl-X_L_), with a concomitant increase in the levels of caspase-3, caspase-9 and cleaved poly-ADP-ribose polymerase (PARP) in SHG-44 after treatment for 48 h (Figure 5). These data showed that PK induced SHG-44 cells apoptosis through a caspase dependent pathway (Figure 6).

## 4. Discussion

Malignant gliomas are the most common primary brain tumors. The patients with malignant gliomas remain poorly responsive to multimodality therapeutic interventions, including surgery, radiotherapy, and chemotherapy [11, 12]. It has been reported that tumor growth is dependent on not only the rate of cellular proliferation, but also that of cell death [13]. Dysregulation of apoptosis contributes directly to tumor development and progression including brain tumors [14–16]. In addition, neoplastic cells' loss of the ability to undergo apoptosis is an important factor that determines the response to treatment with radio- or chemotherapy [17, 18]. 

SHG-44 human glioma cell line was derived from a patient diagnosed with a most malignant and highly invasive grade IV glioma, known as GBM, and is frequently used as a model system to study invasive properties of gliomas in many studies. In the present paper, we demonstrated that PK inhibited the glioma tumor growth and induced the apoptosis of SHG-44 glioma cells. Hoechst 33258 staining and FACS analysis demonstrated that the majority of the glioma cell death is due to apoptosis, and the difference between cell death and apoptosis may result from nonspecific cell death. To gain insight into the molecular mechanism involved in apoptosis by PK, expression of apoptotic-related proteins, Bcl-2, Bcl-X_L_, Bax, and caspase-3, were assessed in SHG-44 glioma cells. We showed that the induction of apoptosis was accompanied by the down-regulation of Bcl-2 gene expression and up-regulation of Bax gene expression, suggesting that alteration of Bcl-2 and Bax is directly or indirectly involved in the apoptotic effect of PK in SHG-44 cells. The expression ratio of proapoptotic and antiapoptotic genes may determine whether a cell lives or dies following an insult [19]. Overexpression of Bcl-2 protects cells from apoptosis following exposure to a number of different proapoptotic stimuli [20], whereas overexpression of Bax renders cells more sensitive to proapoptotic stimuli [21]. Similarly, we also found that PK treatment causes a decreased Bcl-2 expression and increased level of Bax, which may be responsible for the induction of apoptosis in SHG-44 glioma cells. It has been reported that the ICE/caspase family plays a crucial role in apoptosis [22]. In particular, caspase-3 has been shown to be a key component of the apoptotic machinery [23–25]. Our data demonstrated that caspase-3 was activated by PK, indicating that caspase-3 might be one of the critical steps in PK-induced apoptosis.

## 5. Conclusion

This study clearly demonstrates that the water extract of PK strongly inhibits cell proliferation and induces apoptosis in SHG-44 cells. PK induced apoptosis through the activation of caspases-9 and -3 and degradation of PARP. Because apoptosis was regarded as a new target in discovery of anticancer drugs, these results confirm the potential of PK as an agent of chemotherapeutic and cytostatic activity in human glioma cells. Although PK is most commonly used in Chinese medicine, the mechanistic aspects of its effects are still unknown and potential of its bioactive components are yet to be recognized [25, 26]. Thus, evaluation and characterization of the water-soluble active components for discovery of potentially safe glioma-therapeutic phyto-reagents is warranted.

## Figures and Tables

**Table 1:** The composition of “Pingliu Keli” (PK).

Species	Chinese name	Plant part	Origin	Grams	%
*Lycium chinense*	Gou Qi	Seed	Ningxia, China	40	11.1
*Dendrobium officinale*	Shi hu	Stem	Yunnan, China	40	11.1
*Arisaema heterophyllum*	Tian nan xing	Stem	Guizhou, China	40	11.1
*Rhizoma typhonii*	Bai fu zi	Stem tuber	Tibet, China	40	11.1
*Curcuma zedoaria*	E shu	Stem tube	Guangxi, China	40	11.1
*Ligusticum chuanxiong*	Chuan xiong	Root	Sichuan, China	40	11.1
*Buthus martensii Karsch*	Xie zi	Dried body	Shandong, China	40	11.1
*Bombyx mori L*	Jia can	Dried body	Jiangsu, China	40	11.1
*Herba hedyotis diffusae*	She she cao	Leave	Yunnan, China	40	11.1

Total amount				360	100
